# MKL1 defines the H3K4Me3 landscape for NF-κB dependent inflammatory response

**DOI:** 10.1038/s41598-017-00301-w

**Published:** 2017-03-15

**Authors:** Liming Yu, Fei Fang, Xin Dai, Huihui Xu, Xiaohong Qi, Mingming Fang, Yong Xu

**Affiliations:** 1 Key Laboratory of Cardiovascular Disease and Key Laboratory of Human Functional Genomics of Jiangsu Province, Department of Pathophysiology, Nanjing Medical University, Nanjing, China; 2 State Key Laboratory of Nature Medicines, China Pharmaceutical University, Nanjing, China

## Abstract

Macrophage-dependent inflammatory response is considered a pivotal biological process that contributes to a host of diseases when aberrantly activated. The underlying epigenetic mechanism is not completely understood. We report here that MKL1 was both sufficient and necessary for p65-dependent pro-inflammatory transcriptional program in immortalized macrophages, in primary human and mouse macrophages, and in an animal model of systemic inflammation (endotoxic shock). Extensive chromatin immunoprecipitation (ChIP) profiling and ChIP-seq analyses revealed that MKL1 deficiency erased key histone modifications synonymous with transactivation on p65 target promoters. Specifically, MKL1 defined histone H3K4 trimethylation landscape for NF-κB dependent transcription. MKL1 recruited an H3K4 trimethyltransferase SET1 to the promoter regions of p65 target genes. There, our work has identified a novel modifier of p65-dependent pro-inflammatory transcription, which may serve as potential therapeutic targets in treating inflammation related diseases.

## Introduction

A balanced immune response is paramount to the homeostasis whereas deregulated immunity engenders chronic inflammation and is blamed for the pathogenesis of a host of disease states in humans^1^. Regardless of the nature of the stimuli, the twists and turns of cellular immune response invariably settle on a transcriptional network spearheaded by a few evolutionarily conserved transcription factors among which NF-κB is considered the master regulator of pro-inflammatory transcription^2^. To ensure an immune response of desired magnitude, the intensity and duration of NF-κB dependent transcription is tightly controlled. NF-κB tailors pro-inflammatory transcription programs by forging extensive protein-protein interactions with its binding partners that in turn modulate its subcellular localization^3^, post-translational modifications^4^, and/or chromatin structure of its target promoters^5^.

Mounting evidence has highlighted the importance of the epigenetic machinery in fine-tuning NF-κB dependent inflammatory response. Several recent reports have implicated histone H3K4 methyltransferases, including MLL1^6^, MLL4/WBP7^7^, and SET7/9^8^, as essential for the transactivation of a subset of NF-κB target genes. Alternatively, SMYD5 mediated methylation of H4K20 has been proposed as a repressive signature that halts the transcription of pro-inflammatory genes downstream of TLR4 signaling^9^. Therefore, a consensus seems to be building up that chromatin structure is a pivotal determinant of NF-κB driven transcription.

Rel-A/p65 is the most well studied member of the NF-κB family primarily forming a heterodimer with p50 that represents a key node in the intertwined inflammatory circuit^10^. Our previous publication has characterized a physical interaction between p65 and megakyrocytic leukemia 1 (MKL1)^11^. MKL1, also known as myocardin related transcription factor A (MRTF-A), was initially identified as a co-factor for SRF involved in the transcriptional regulation of smooth muscle contraction genes^12^. Unlike myocardin that shows a muscle-restricted expression pattern, MKL1 is universally expressed^12^. MKL1 regulates transcription and, by extension, cellular processes by interacting with sequence-specific transcription factors including SMAD3^13^, Sp1^14^, AP-1^15^, STAT3^16^, and TEAD^17^. Recently, a number of independent investigations have pitched MKL1 as a potential stress protein that links specific transcription event(s) to cellular adaptation to cardiovascular risk factors^18–20^. Based on these findings, we asked whether and if so how MKL1 modifies p65 dependent pro-inflammatory transcriptional program. Our data presented here suggest that MKL serves as a coordinator defining the epigenetic landscape for p65.

## Results

### MKL1 potentiates NF-κB-dependent transcription

**Figure 1:**
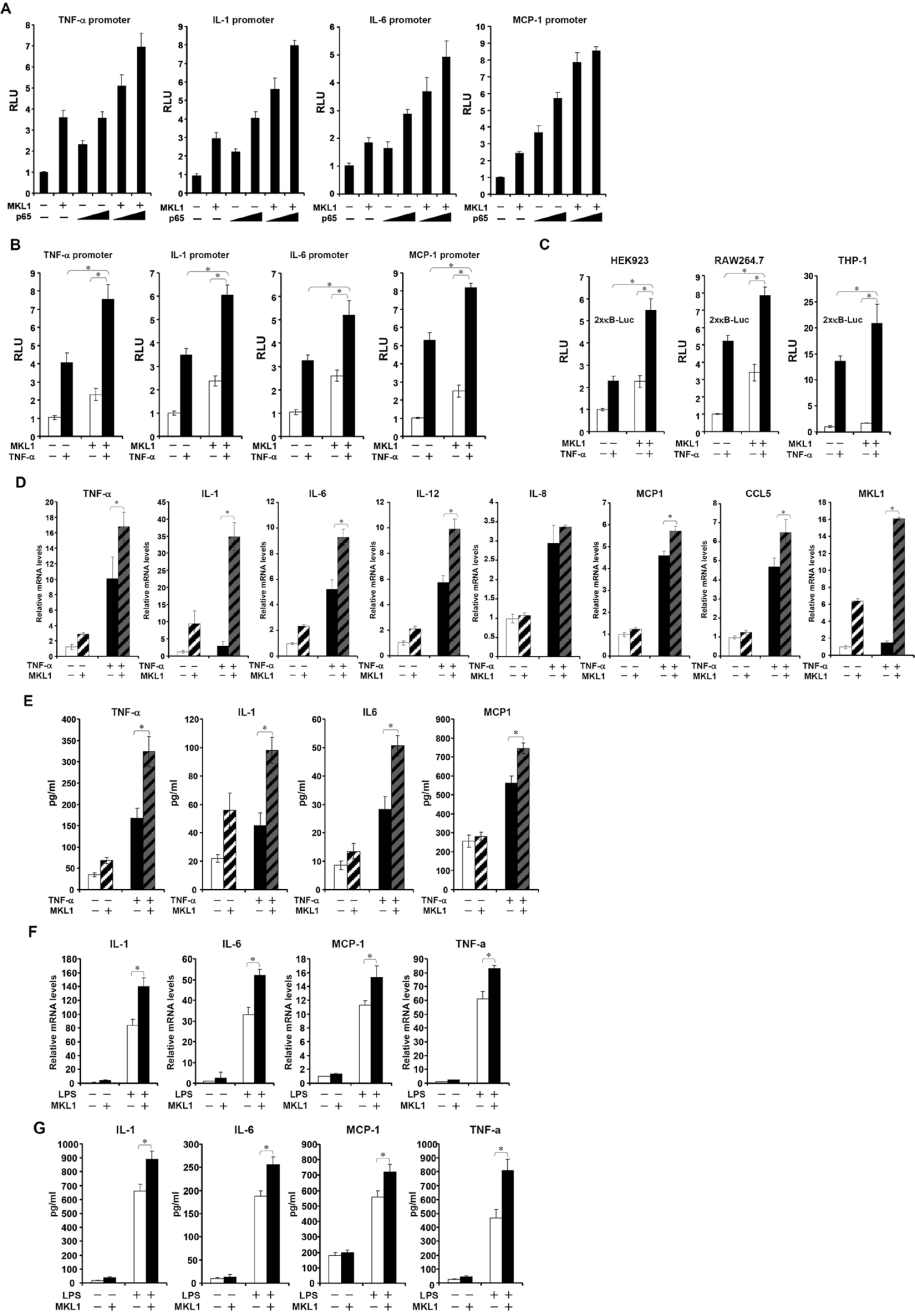
MKL1 enhances Rel-A/p65 dependent transcription. (**A**) FLAG-MKL1 and V5-p65 were co-transfected into HEK293 cells with indicated promoter constructs. Data are expressed as relative luciferase unit (RLU). (**B**) FLAG-MKL1 was co-transfected into HEK293 cells with indicated promoter constructs followed by treatment with TNF-α (10 ng/ml) for 6 hours. Data are expressed as RLU. (**C**) FLAG-MKL1 was co-transfected into cells with a generic κB reporter construct followed by treatment with TNF-α (10 ng/ml) for 6 hours. Data are expressed as RLU. (**D**,**E**) THP-1 cells were transfected FLAG-MKL1 followed by treatment with TNF-α (10 ng/ml) for 6 hours. mRNA (**D**) and protein (**E**) levels of pro-inflammatory mediators were measured by qPCR and ELISA. (**F**,**G**) THP-1 cells were transfected FLAG-MKL1 followed by treatment with LPS (100 ng/ml) for 6 hours. mRNA (**F**) and protein (**G**) levels of pro-inflammatory mediators were measured by qPCR and ELISA.

To verify whether MKL1 serves as a functional partner for p65 and plays a role in pro-inflammatory transcription, we performed reporter assays. We transfected into HEK293 cells a series of promoter luciferase constructs for established p65 target genes (Fig. 1A, S1A,B). With the exception of IL-8, MKL1 markedly enhanced the induction of promoter activities by p65. MKL1 also augmented the induction of promoter activities by TNF-α (Fig. 1B, S1C); MKL1 exerted little effect on the basal promoters, suggesting that MKL1 probably does not influence basal transcription (Fig. S1D). Consistently, MKL1 activated a generic κB reporter with TNF-α in HEK293 cells and murine and human macrophages (Fig. 1C). In contrast, MKL2, a closely related protein, exhibited much weaker activity on the κB reporter (Fig. S1E). Of note, other members of the NF-κB family did not interact with MKL1 either physically (Fig. S1F,G) or functionally (Fig. S1H).

Next, we examined the production of endogenous pro-inflammatory mediators under the influence of MKL1. Over-expression of a constitutively active (CA) form of MKL1 increased the synthesis and release of pro-inflammatory mediators from TNF-α treated THP-1 cells (Fig. 1D,E). We also evaluated the effect of MKL1 over-expression on the synthesis of pro-inflammatory cytokines by LPS. As shown in Fig. 1E and F, MKL1 markedly enhanced the production of IL-1, TNF-α, IL-6 and MCP-1 in THP-1 cells by LPS﻿. Of note, both TNF-α (Fig. S2A and B) and LPS (Fig. S3A and B) treatment modestly but significantly elevated the expression levels of MKL1 in THP-1 cells. TNF-α and LPS stimulation also promoted the nuclear translocation of MKL1 in THP-1 cells as evidenced by cell fractionation/immunoblotting (Figs S2C and S3C). Thus, MKL1 is able to enhance cellular response to pro-inflammatory stimuli by potentiating NF-κB/p65 dependent transcription.

### MKL1 deficiency cripples NF-κB-dependent transcription

**Figure 2:**
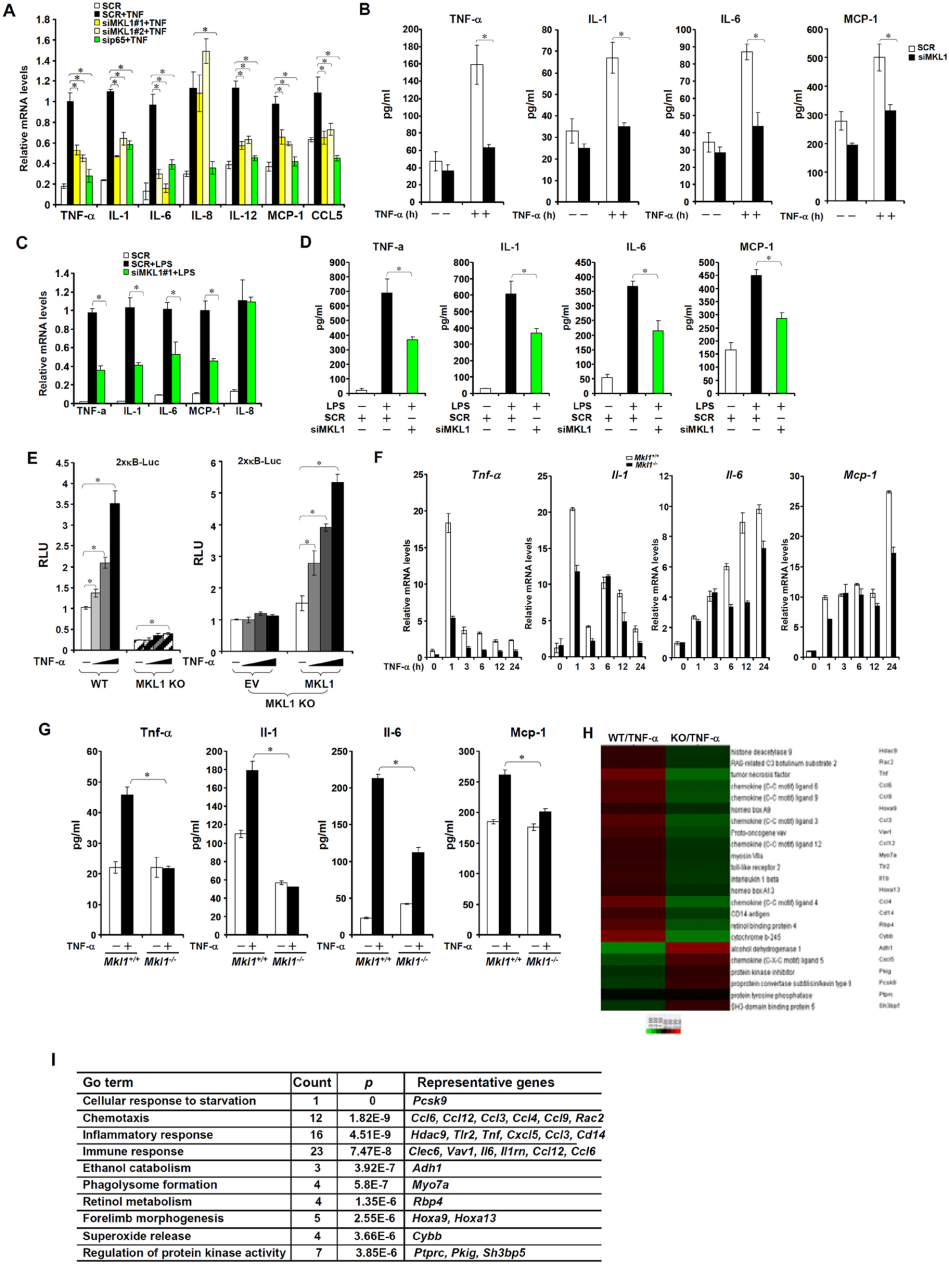
MKL1 deficiency dampens Rel-A/p65 dependent transcription. (**A**,**B**) THP-1 cells were transfected with indicated siRNAs followed by treatment with TNF-α (10 ng/ml) for 6 hours. mRNA (**A**) and protein (**B**) levels of pro-inflammatory mediators were measured by qPCR and ELISA. (**C**,**D**) THP-1 cells were transfected with indicated siRNAs followed by treatment with LPS (100 ng/ml) for 6 hours. mRNA (**C**) and protein (**D**) levels of pro-inflammatory mediators were measured by qPCR and ELISA. (**E**) WT or MKL KO MEF cells were transfected with a generic κB reporter construct followed by treatment with TNF-α (10 ng/ml) for 6 hours. Data are expressed as RLU. (**F**–**I**) MEF cells were treated with TNF-α (10 ng/ml). mRNA (**F**) and protein (**G**) levels of pro-inflammatory mediators were measured by qPCR and ELISA. (**H**) Heat map and (**I**) Go analysis.

Next, we employed several different strategies to tackle the question whether MKL1 deficiency would dampen NF-κB dependent transcription. We first used small interfering RNA (siRNA) to knock down MKL1 expression in THP-1 cells (Fig. S8 for efficiency). As shown in Fig. 2A and B, MKL1 silencing abrogated the induction of endogenous pro-inflammatory mediators in the presence of TNF-α. Similarly, MKL1 knockdown also attenuated LPS-induced synthesis of pro-inflammatory mediators in THP-1 cells (Fig. 2C,D). In keeping with these observations, TNF-α failed to evoke as strong a response of the κB reporter in MKL1-null mouse embryonic fibroblast (MEF) cells as in wild type (WT) cells, a phenotype which was corrected by the re-introduction of exogenous MKL1 (Fig. 2E). To affirm the notion that MKL1 is essential for cellular response to pro-inflammatory stimuli, MEF cells (Fig. 2F,G) and primary peritoneal macrophages (Fig. S4A,B) were isolated from wild type (*Mkl* ^+/+^) or MKL1 knockout (*Mkl1* ^−/−^) and treated with TNF-α followed qPCR and ELISA analyses to examine the expression of endogenous pro-inflammatory mediators. MKL1 loss-of-function invariably decreased the production of pro-inflammatory mediators in both cell types examined in this study.

To gain insight from a genomewide perspective how MKL1 deficiency would affect cellular response to TNF-α, we performed microarray analysis using *Mkl* ^+/+^ or *Mkl1* ^−/−^ MEF cells. MKL1 ablation profoundly altered the transcriptome of MEF cells in response to pro-inflammatory stimuli (Fig. 2H). A number of genes involved in cellular inflammatory response including *Tnf*, *Il-1*, *Ccl4, Ccl9, Tlr2, and Cxcl5* were found to be down-regulated in *Mkl1* ^−/−^ MEF cells when compared to *Mkl* ^+/+^ MEF cells. Indeed, Go analysis of expression profile (Fig. 2I) indicated that several pathways related to innate immunity/inflammation were affected by MKL1 deficiency. “Chemotaxis” (ranked #2), “inflammatory response” (ranked #3), and “immune response” (ranked #4) were among the top five hits. Therefore, MKL1 is essential for NF-κB-dependent pro-inflammatory transcription.

### A dynamic interaction between MKL1 and p65

**Figure 3:**
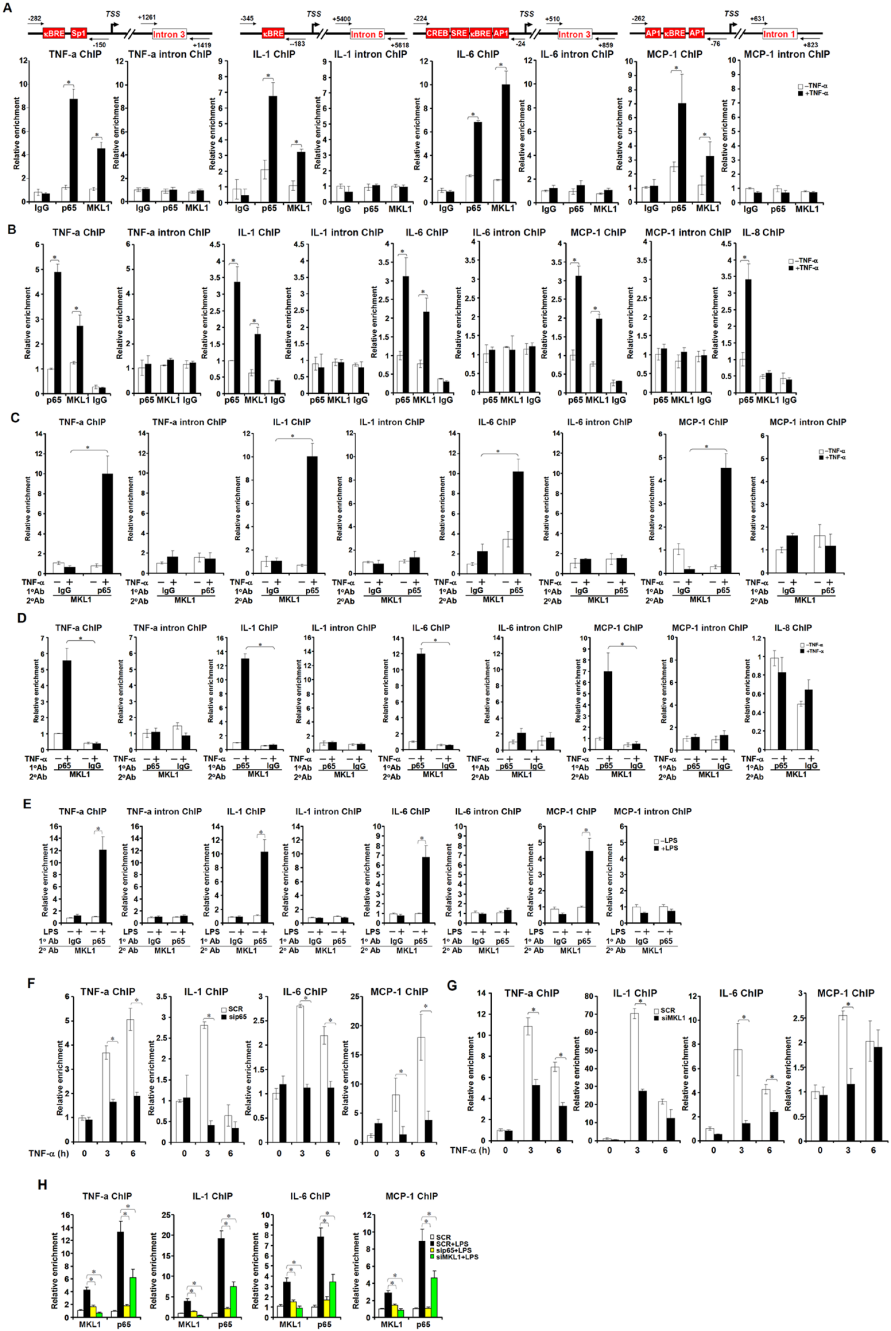
A reciprocal interplay between MKL1 and p65 on the chromatin. (**A**,**B**) THP-1 cells (**A**) or differentiated primary human macrophages (**B**) were treated with TNF-α (10 ng/ml) for 6 hours. ChIP assays were performed with indicated antibodies. Precipitated DNA was amplified with indicated primers shown with conserved binding motifs. κBRE, NF-κB response element; SRE, serum response element; TSS, transcription start site (**C**,**D**) THP-1 cells (**C**) and differentiated primary human macrophages (**D**) were treated with or without TNF-α (10 ng/ml) for 3 hours. Re-ChIP assays were performed with indicated antibodies. (**E**) THP-1 cells were treated with or without LPS (100 ng/ml) for 6 hours. Re-ChIP assays were performed with indicated antibodies. (**E**,**F**) THP-1 cells were transfected with indicated siRNA followed by treatment with TNF-α (10 ng/ml) for 6 hours. ChIP assays were performed with anti-MKL1 (**E**) or anti-p65 (**F**). (**H**) THP-1 cells were transfected with indicated siRNA followed by treatment with LPS (100 ng/ml) for 6 hours. ChIP assays were performed with anti-MKL1 or anti-p65.

MKL1 is recruited to target promoters via protein-protein interactions. In response to TNF-α treatment, there was an increased occupancy of MKL1 on gene promoters along with p65 in THP-1 cells as evidenced by ChIP assay (Figs 3A, S5A). Similarly, we also observed enhanced binding of MKL1 on NF-κB target promoters in freshly isolated and differentiated human peripheral blood macrophages (HPBMs, Fig. 3B). More importantly, the formation of a p65-MKL1 complex, as examined by Re-ChIP assay, was enhanced by TNF-α in both THP-1 cells (Fig. 3C) and HPBMs (Fig. 3D). LPS treatment also promoted the assembly of the p65-MKL1 complex on the promoter regions of pro-inflammatory mediators (Fig. 3E). In keeping with a previous report^11^, p65 silencing (Fig. S8 for efficiency) essentially neutralized MKL1 binding in response to TNF-α stimulation (Fig. 3F). Reciprocally, MKL1 knockdown altered the kinetics of p65 occupancy: p65 binding to its target promoters following TNF-α stimulation was impaired in the absence of MKL1 (Fig. 3G). Similar observations were made in LPS-treated THP-1 cells : while MKL1 recruitment to pro-inflammatory gene promoters required p65, p65 binding was impaired without MKL1 (Fig. 3H).

We also performed ChIP assays in BMDMs (Fig. S5B) and MEFs (Fig. S5C) and we found that p65 binding on its target promoters was much weaker in MKL1 deficient cells than in WT cells. Additionally, MKL1 deficiency negatively impacted the nuclear accumulation of p65 as assessed by cell fractionation/Western blotting (Fig. S5D,E), immunofluorescence staining (Fig. S5F), and gel shift (Fig. S5G) assays; these data all suggest an essential role for MKL1 in maintaining/sustaining the trans-localization and target binding of p65 in response to TNF-α. Together, this line of evidence portrays MKL1 as an integral part of the p65-centered complex responsible for modulating the inflammatory response.

### MKL1 deficiency tempers inflammatory response in mice

**Figure 4:**
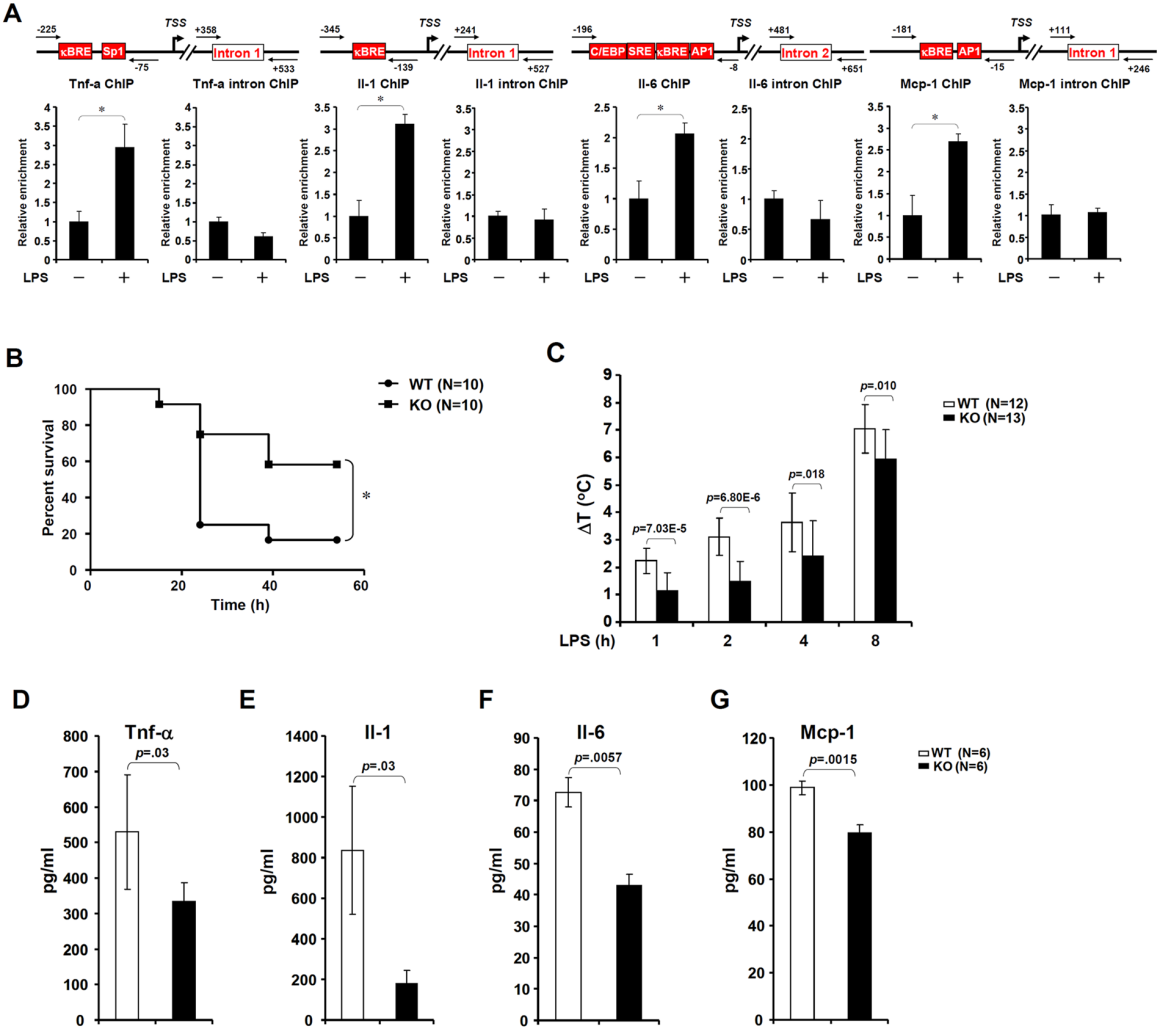
MKL1 deficiency cripples inflammatory response in mice. (**A**) C57/BL6 mice were injected with LPS (25 mg/kg). Peritoneal macrophages were isolated 6 hours after injection and ChIP assays were performed with anti-MKL1. (**B**,**C**) Sex- and age-matched WT and MKL1 KO male mice were injected with LPS (25 mg/kg). Survival (**B**) and body temperature (**C**) were monitored following injection. N = 10 mice for each group. (**D**–**G**) Mice were sacrificed 4 hours after injection. Circulating levels of pro-inflammatory mediators were measured by ELISA. N = 6 mice for each group.

Next, we sought to evaluate the impact of MKL1 deficiency on inflammation response in a classic animal model of systemic inflammation, namely LPS-induced endotoxic shock. As shown in Fig. 4A, peritoneal macrophages isolated from mice with endotoxic shock exhibited stronger MKL1 binding on the promoters of pro-inflammatory genes than the control mice. More importantly, MKL1-null mice exhibited markedly improved survival rate following intraperitoneal injection of LPS (Fig. 4B). Hypothermia, a common pathophysiological alteration associated with shock, was also significantly ameliorated in MKL1 deficient mice (Fig. 4C). These changes were paralleled by diminished levels of pro-inflamamtory mediators in the circulation (Fig. 4D–G). Together, these data suggest that MKL1 is imperative for a full-fledged inflammatory response *in vivo*.

### MKL1 influences the chromatin structure of pro-inflammatory genes

**Figure 5:**
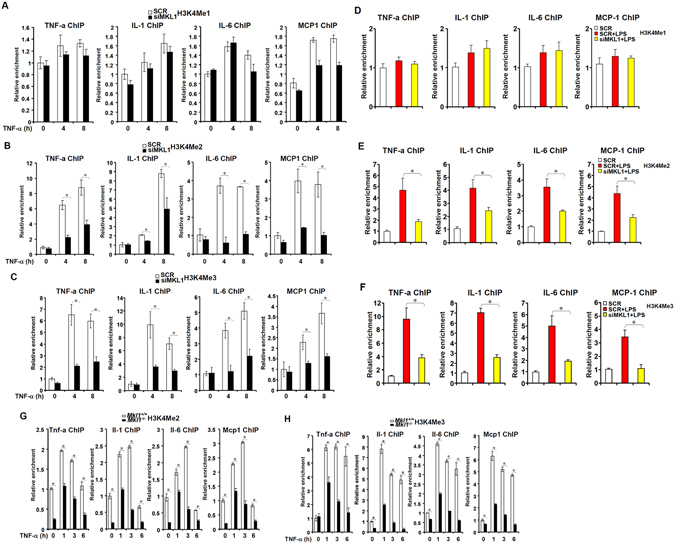
MKL1 influences the chromatin structure of pro-inflammatory genes. (**A**–**C**) THP-1 cells were transfected with indicated siRNA followed by treatment with TNF-α (10 ng/ml). ChIP assays were performed with anti-monomethyl H3K4 (**A**), anti-dimethyl H3K4 (**B**), and anti-trimethyl H3K4 (**C**). (**D**–**F**) THP-1 cells were transfected with indicated siRNA followed by treatment with LPS (100 ng/ml) for 6 hours. ChIP assays were performed with anti-monomethyl H3K4 (**D**), anti-dimethyl H3K4 (E), and anti-trimethyl H3K4 (**F**). (**G**,**H**) WT or KO BMDMs were treated with TNF-α (10 ng/ml). ChIP assays were performed with anti-dimethyl H3K4 (**G**) and anti-trimethyl H3K4 (**H**).

MKL1 is known to be allied with the epigenetic machinery in regulating transcription^11, 21–24^. Therefore, we asked whether MKL1 deficiency would lead to the disruption of an active chromatin structure, marked by histone H3K4 methylation, in macrophages treated with TNF-α. Indeed, depletion of MKL1 prevented the enrichment of dimethylated and trimethylated but not monomethylated H3K4 following TNF-α stimulation (Fig. 5A–C). Likewise, we found that MKL1 was necessary for LPS-induced accumulation of di- and tri-methylated, but not monomethylated, H3K4 on the promoter regions of pro-inflammatory cytokines (Fig. 5D–F). Furthermore, the increase in H3K4Me2 and H3K4Me3 levels surrounding the promoter regions of p65 target genes in response to TNF-α treatment was attenuated in *Mkl1* ^−/−^ BMDMs compared to *Mkl1* ^+/+^ BMDMs (Fig. 5G,H). We also examined the effects of MKL1 silencing on histone acetylation surrounding the pro-inflammatory gene promoters. Consistent with previous reports^15, 25^, MKL1 silencing was associated with the loss of acetylated H3 from the gene promoters although acetylation of specific lysine residues showed varied dependence on MKL1 (Fig. S6).

**Figure 6:**
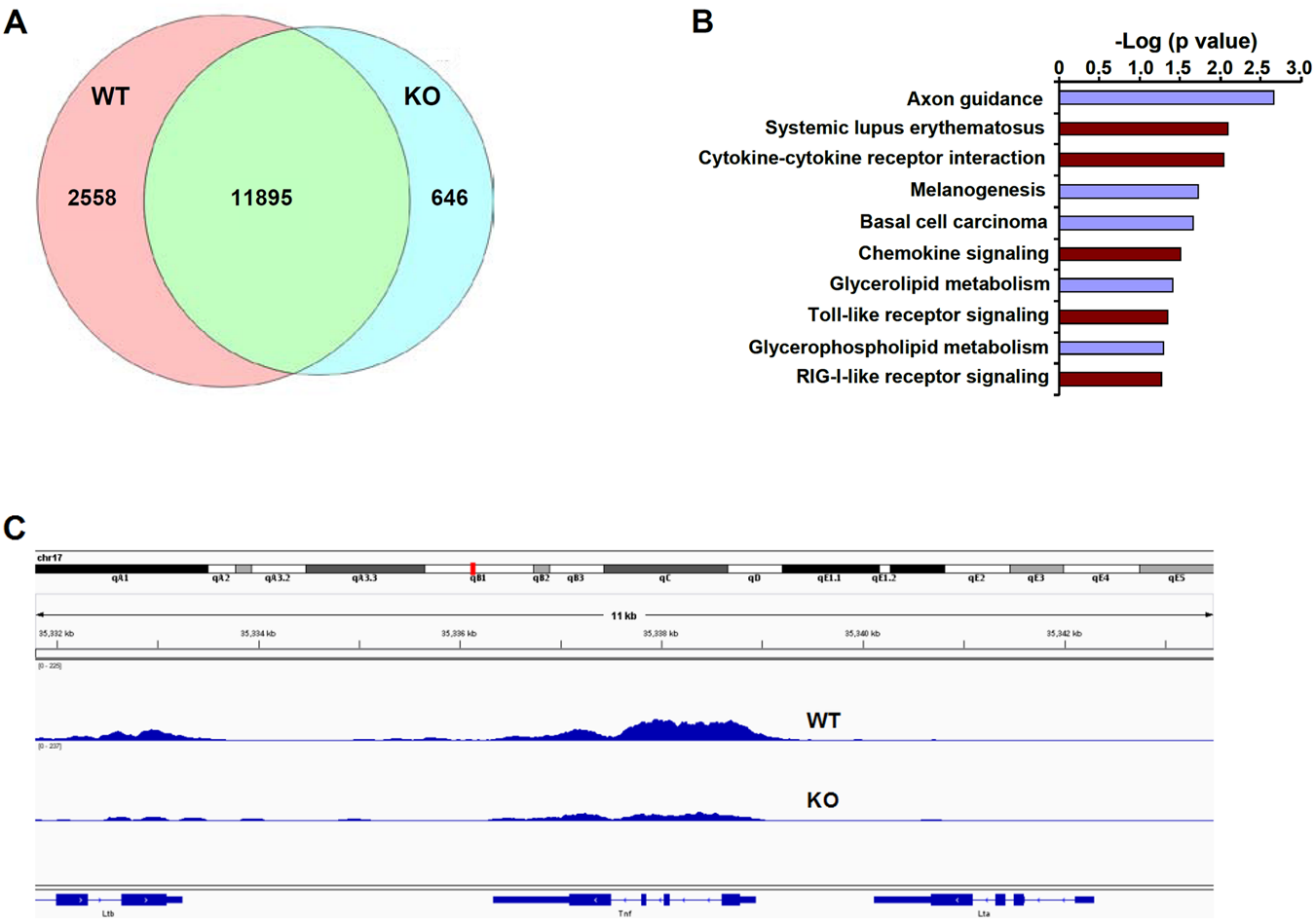
MKL1 influences global H3K4Me3 levels in BMDMs. Wild type (WT) or MKL1 deficient (KO) BMDMs were treated with TNF-α (10 ng/ml) for 3 hours. ChIP was performed with anti-H3K4Me3. (**A**) Venn diagram illustrating the overlap of binding peaks identified in WT and KO BMBM ChIP-seq data sets. (**B**) Pathway analysis of significantly enriched H3K4Me3 tags found within ±2 kb relative to the TSS of the nearest gene. (**C**) Graphical view of H3K4Me3 ChIP-seq binding peaks (tag densities) around the TSS of TNF-α.

In order to extrapolate the aforementioned findings to a larger scale, we performed ChIP-seq analysis of H3K4Me3 levels in WT and MKL1 deficient (KO) BMDMs. With a false discovery rate of 0.1%, a total of 14453 unique peaks were called for the WT BMDMs whereas a total of 12251 unique peaks were identified for the MKL1 KO BMDMs. Most of the H3K4Me3 peaks in both WT (Fig. S7A) and KO (Fig. S7B) cells were found to surround the transcription start site (TSS), consistent with the well-known role for H3K4Me3 as a maker of transcriptional activation. Although binding peaks showed a large overlap in WT and KO cells, there were significant numbers of unique peaks found only in WT cells (2558, Fig. 6A). Pathway analyses of WT-specific peaks suggest that a large portion of the genes around these peaks were involved in immune response (Fig. 6B); functional annotation found that such inflammatory response-related pathways as “systemic lupus erythematosus” (ranked #2), “cytokine-cytokine receptor interaction” (ranked #3), “chemokine signaling” (ranked #6), “toll-like receptor signaling” (ranked #8), and “RIG-1-like receptor signaling” (ranked #10) were among the top 10 hits. We also found that peaks surrounding several unique pro-inflammatory mediator genes including TNF-α (Fig. 6C), CCL3 (Fig. S7C), CCL4 (Fig. S7D), and CCL9 (Fig. S7E) were attenuated in KO cells when compared to WT cells. Together, these data confirm the role of MKL1 as a global modulator of histone modification and chromatin structure in inflamed macrophages.

### MKL1 recruits SET1 to activate the transcription of pro-inflammatory mediators

**Figure 7:**
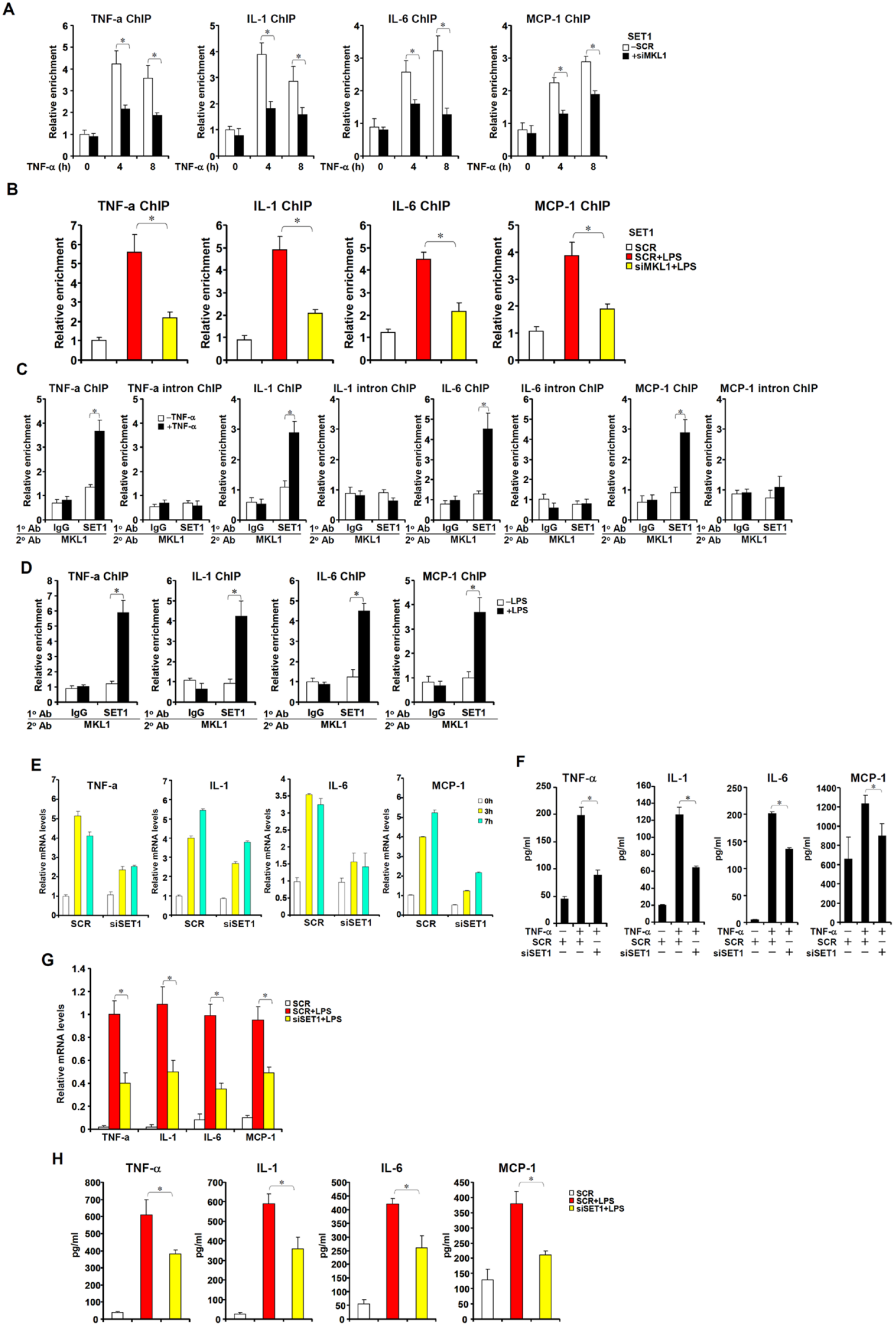
MKL1 recruits SET1 to activate the transcription of p65 target genes. (**A**) THP-1 cells were transfected with indicated siRNA followed by treatment with TNF-α (10 ng/ml) and harvested at indicated time points. ChIP assays were performed with anti-SET1. (**B**) THP-1 cells were transfected with indicated siRNA followed by treatment with LPS (100 ng/ml) for 6 hours. ChIP assays were performed with anti-SET1. (**C**) THP-1 cells were treated with or without TNF-α (10 ng/ml) for 3 hours. Re-ChIP assays were performed with indicated antibodies. (**D**) THP-1 cells were treated with or without LPS (100 ng/ml) for 6 hours. Re-ChIP assays were performed with indicated antibodies. (**E**) THP-1 cells were transfected with indicated siRNA followed by treatment with TNF-α (10 ng/ml) and harvested at indicated time points. mRNA levels of pro-inflammatory mediators were measured by qPCR. (**F**) THP-1 cells were transfected with indicated siRNA followed by treatment with TNF-α (10 ng/ml) for 6 hours. Protein levels of pro-inflammatory mediators were measured by qPCR. (**G**,**H**) THP-1 cells were transfected with indicated siRNA followed by treatment with LPS (100 ng/ml) for 6 hours. mRNA (**G**) and protein (**H**) levels of pro-inflammatory mediators were measured by qPCR and ELISA.

In mammals, histone H3K4 trimethylation is mediated by the COMPASS complex, of which SET1 is the core catalytic component^26^. In response to either TNF-α (Fig. 7A) or LPS (Fig. 7B) stimulation, there was increased occupancy of SET1 on the promoter regions of p65 target genes. The binding of SET1 to p65 target promoters appeared to depend on, at least in part, MKL1 as MKL1 knockdown significantly attenuated SET1 recruitment (Fig. 7A and B). Furthermore, TNF-α (Fig. 7C) or LPS (Fig. 7D) treatment promoted the interaction between MKL1 and SET1 on the promoter regions of p65 target genes as evidenced by Re-ChIP assays. When endogenous SET1 was depleted by siRNA (see Fig. S7 for knockdown efficiency), the synthesis of pro-inflammatory mediators induced by either TNF-α (Fig. 7E,F) or LPS (Fig. 7G,H) was significantly attenuated. Together, we propose that MKL1 may regulate the epigenetic activation of p65-dependent pro-inflammatory transcription in THP-1 cells via recruiting the H3K4 trimethyltransferase SET1.

## Discussion

Previous studies have detailed potential roles of MKL1 in cardiac myocyte^19^, smooth muscle cells^27^, epithelial cells^13^, fibroblast cells^14, 28^, and endothelial cells^11, 24^. By providing the first snapshot of MKL1 function in macrophages, the current report greatly expands our understanding of MKL1 as an epigenetic modulator of NF-κB-dependent pro-inflammatory transcription. Extensive ChIP profiling and ChIP-seq analyses clearly demonstrate that MKL1 is able to elicit profound changes on NF-κB target promoters. In light of previous reports, based on single-gene analysis, which implicate MKL1 in the epigenetic regulation of transcription^11, 21, 23, 29^, our data reinforce the notion that MKL1 is an integral part of the cellular network bridging the epigenetic machinery to sequence-specific transcription factors.

Our data demonstrate a complex and dynamic interplay between MKL1 and NF-κB/p65. On the one hand, NF-κB depletion blocked MKL1 recruitment (Fig. 3E). Therefore, our data indicate that MKL1-induced transactivation of pro-inflammatory transcription, to a large extent, relies on NF-κB. However, the participation of sequence-specific transcription factors other than NF-κB cannot be completely excluded at this point. For instance, the promoters of the genes examined here contain binding elements for several sequence-specific factors known to interact with MKL1 including AP-1, SRF, and Sp1 (Fig. S1A). Two recent studies examining genome-wide binding patterns of MKL1 in fibroblast^30^ and macrophages^31^ confirm that in addition to NF-κB, MKL1 may co-occupancy the DNA with SRF, AP-1, and Sp1 although it is not clear how the dynamic changes under pro-inflammatory stimuli (e.g., TNF-α). Additional studies are warranted to solve this lingering issue.

Of intrigue, the affinity of NF-κB for several of its target promoters seems to be significantly weakened in the absence of MKL1, indicating that MKL1 might reciprocally regulate NF-κB activity. A couple of recent investigations have alluded to the preferential localization of p65 to chromatin regions enriched with modified histones (e.g., H3K3Me1 and acetyl H3K9)^32^. It is thus conceivable that MKL1 may interfere with the binding of NF-κB via influencing specific histone modifications. Alternatively, in the absence of MKL1 p65 tends to bind to chromatin less stably and as a result more susceptible to be removed from the nucleus. Therefore, the observed decrease in nuclear p65 reflects a tilted dynamics of p65 import/export, i.e. an accelerated p65 outflow rather than decelerated p65 inflow. Finally, several independent investigations have shown that p65 translocation and activation are intimately coupled to cytoskeleton re-organization^33, 34^. MKL1 has long been hailed as imperative in maintaining cellular motile function^35^. In cells lacking MKL1, altered cytoskeleton arrangements may prevent the nuclear import of p65. Indeed, our report here fits with the newly emerged consensus that there is an intimate connection between cytoskeleton dynamics and epigenetic regulation of inflammatory responses^36^. Of note, a couple of recent papers suggest that MKL1 could act an anti-inflammation transcription factor. Wang *et al.* reported that MKL1 mediates the anti-inflammatory effects of BMP in pulmonary vascular smooth muscle cells^37^. Hayashi *et al.* reported that MKL1 inhibits p65 activity in aortic endothelial cells^38^. It thus appears the MKL1 may regulate inflammatory response in a cell-specific manner. Further investigations are required to solidify the role of MKL1 as a central coordinator of this important pathobiological process.

Owing to the universal expression pattern of MKL1, the phenotypes of MKL1 deficient mice as reported here should be interpreted with caution. We have observed that MKL1 mediates the stimulation of adhesion molecules (CAM) in endothelial cells in response to pro-inflammatory stimuli^11, 39^. CAM-dependent interactions between endothelium and circulating leukocytes are essential for the inflammatory response associated with endotoxic shock^40^. Therefore, attenuated inflammation in MKL1 KO mice could be attributed to disrupted endothelium-leukocyte interaction as a result of crippled CAM induction rather than reduced synthesis of pro-inflammatory mediators in macrophages. Future investigations should aim at sorting out the cell-autonomous role for MKL1.

We show here that MKL1 influences histone H3K4 trimethylation likely through the recruitment of an H3K4 trimethyltransferase SET1 (Fig. 7). Indeed, SET1 depletion attenuates the induction of pro-inflammatory mediators by either TNF-α or LPS (Fig. 7). There are, however, several caveats regarding this model. First, H3K4 trimethylation status across the genome reflects the dynamics of not only methyltransferases (e.g., SET1) but demethylases. In mammals, several H3K4Me3 demethylases including RBP2, PLU1, and SMCX have been identified^41^. It is entirely possible that MKL1 might, via some unknown mechanism, expels one or several of these demethylases from the promoters of pro-inflammatory genes to activate transcription. Second, transcription activation is associated with the accumulation of active histone marks (e.g., H3K4Me3) and the disappearance of repressive histone marks (e.g., H3K9Me2). Previously, it has been shown that MKL1 recruits an H3K9Me2 demethylase Jmjd1a to remodel the chromatin surrounding the promoters of smooth muscle contraction genes thereby activating transcription^22^. The possibility that MKL1 simultaneously interacts with and recruits methyltransferases that catalyze active histone modifications and demethylases that remove repressive histone modifications should be further explored in the future. Finally, in addition to influencing histone methylation MKL1 could potentially regulate histone acetylation on the promoters of p65 target genes (Fig. S5). This observation is consistent with several previous reports that describe the interactions between MKL1 and histone acetyltransferases (e.g., p300) to activate transcription in a number of different cells^23, 42, 43^. A recent report by Tang *et al.* suggests a potential crosstalk between SET1 and p300 to synergistically activate the transcription of p53 target genes^44^. Therefore, it is likely that MKL1 could function as a moderator bridging the communications between different histone modifying enzymes.

Of intrigue, expression and nuclear enrichment of MKL1 seem to be similarly influenced by TNF-α and LPS stimulation. Both TNF-α and LPS have been shown to alter the cytoskeletal dynamics in macrophages to promote inflammation^33, 34, 45^. Therefore, it is likely that the observed effects of TNF-α and LPS on MKL1 expression and activity can be perceived as secondary to the remodeling of cytoskeleton. In a similar vein, MKL1, as a major mediator of cytoskeleton revamping^35^, could potentially link multiple signaling pathways, regardless of the extracellular stimuli, to the same set of transcriptional events in the nucleus. Alternatively, MKL1, by shaping up the chromatin structure, may dictate the transcriptional come of inflammatory response invoked by different stimuli.

Excessive inflammation is blamed for a wide range of cardiovascular diseases^46^. Therefore, a question of critical importance looming large is how the current findings can be translated into potential therapeutic solutions. Transcriptional modulators have long been considered notoriously difficult to drug though there have been a few successes^47, 48^. Our data indicate that MKL1 relies on the engagement of the epigenetic machinery to drive a pro-inflammatory agenda in macrophages. Conceivably, any molecule that can specifically block MKL1 from enlisting the epigenetic machinery, a process highlighted here as essential for MKL1 to activate pro-inflammatory transcription, would tune down cellular inflammation and hence be worth pursuing as a potential solution for the intervention of inflammation-related diseases.

## Materials and Methods

### Cell culture

Human monocytic/macrophage-like cells (THP-1, ATCC) were maintained in DMEM supplemented with 10% FBS. Murine primary peritoneal macrophages and bone-derived macrophages were isolated and cultured as described before^49^. Primary human peripheral blood monocytes were isolated and differentiated as described before^50^.

### Plasmids, transfection, and reporter assay

Expression constructs for MKL1, p65, as well as promoter constructs have been described before^11, 51–58^. siRNA sequences are listed in Supplemental Table 1. For alidation of siRNA mediated knockdown efficiencies, see Supplementary Figure 5. Transient transfections were performed with Lipofectamine LTX (Invitrogen). Luciferase activities were assayed 24–48 hours after transfection using a luciferase reporter assay system (Promega). Experiments were routinely performed in triplicate wells and repeated three times.

### Mice

All animal protocols were approved the intramural Ethics Committee on Animal Studies of Nanjing Medical University and in accordance with the NIH Guidelines for the Care and Use of Laboratory Animals. MKL1 deficient mice have been described previously^59^. To induce endotoxic shock, mice were injected intraperitoneally with LPS (25 mg/kg). Rectal temperature was monitored by a portable digital rodent thermometer (Harvard Apparatus).

### Gel shift assay

Nuclear proteins from wild type or MKL1 deficient MEF cells extracted using a commercially available kit (NE-PER Kit, Pierce). Gel shift assay was performed with 5 μg of nuclear extracts and biotin-labeled DNA probe harboring consensus NF-κB response element (5′-AG*TTGAGGGGACTT*TCCCAGGC-3′) using a LightShift chemiluminescent Kit (Pierce) following the vendor's recommendations.

### ChIP and Re-ChIP assay

ChIP assays were performed essentially as described before^39, 60^. Aliquots of lysates containing 200 μg of nuclear protein were used for each immunoprecipitation reaction with anti-MKL1, anti-p65 (Santa Cruz), anti-SET1 (Bethyl Laboratories), anti-monomethyl H3K4, anti-dimethylated H3K4, and anti-trimethylated H3K4 (Millipore/Upstate). For re-ChIP, immune complexes were eluted with the elution buffer (1% SDS, 100 mM NaCO_3_), diluted with the re-ChIP buffer (1% Triton X-100, 2 mM EDTA, 150 mM NaCl, 20 mM Tris pH 8.1), and subject to immunoprecipitation with a second antibody of interest. Precipitated genomic DNA was amplified by real-time PCR with primers listed in Supplemental Table 2.

### ChIP-sequencing and data analysis

ChIP-seq and data processing were performed essentially as described before^61, 62^. Visualization of ChIP-seq results was achieved by uploading custom tracks onto the University of California, Santa Cruz genome browser. Reads were aligned using the Bowtie aligner. Only tags that map uniquely to the genome were considered for further analysis. Subsequent peak calling and motif analysis were conducted using HOMER. Peaks are annotated to gene products by identifying the nearest RefSeq transcriptional start site. Visualization of ChIP-seq results was achieved by uploading custom tracks onto the University of California Santa Cruz genome browser.

### RNA extraction and real-time PCR

RNA was extracted using an RNeasy RNA isolation kit (Qiagen). Reverse transcriptase reactions were performed using a SuperScript First-strand synthesis system (Invitrogen). Real-time PCR reactions were performed on an ABI STEPONE Plus (Life Tech) with primers and Taqman probes purchased from Applied Biosystems.

### Microarray analysis

Total RNA extracted from wild type (WT) or MKL1 deficient (KO) MEF cells was isolated using the RNeasy kit and quality was assessed using the Agilent Bioanalyzer and prepared for hybridization to Illumina mouse gene expression arrays according to standard Illumina protocols. Normalization and identification of differentially expressed genes were performed using VAMPIRE at http://sasquatch.ucsd.edu/vampire/.

### Immunofluorescence microscopy

Formaldehyde fixed cells were blocked with 5% BSA, and incubated with indicated primary antibodies overnight. After several washes with PBS, cells were incubated with appropriate secondary antibodies (Jackson) for 30 minutes. DAPI (Sigma) was added and incubated with cells for 5 minutes prior to observation. Immunofluorescence was visualized on a co-focal microscope (LSM 710, Zeiss). For quantification, 50~100 cells in triplicate wells were counted for each condition.

### Statistical Analysis

One-way ANOVA with post-hoc Scheffe analyses were performed using an SPSS package. *p* values smaller than 0.05 were considered statistically significant (*).

## Electronic supplementary material

supplementary figures and tables
