# Supplementary material for: MKL1 defines the H3K4Me3 landscape for NF-κB dependent inflammatory response
Source: Sci Rep. 2017 Mar 15;7:191. doi: 10.1038/s41598-017-00301-w (PMC5428227; doi:10.1038/s41598-017-00301-w)
Supplement: Supplementary file 1 — supplementary figures and tables [file 41598_2017_301_MOESM1_ESM.pdf]

Liming Yu, Fei Fang, Xin Dai, Huihui Xu, Xiaohong Qi, Mingming Fang, Yong Xu:

MKL1 defines the epigenetic landscape for NF-κB dependent inflammatory response

Supplemental figures: 8

Supplemental tables: 2

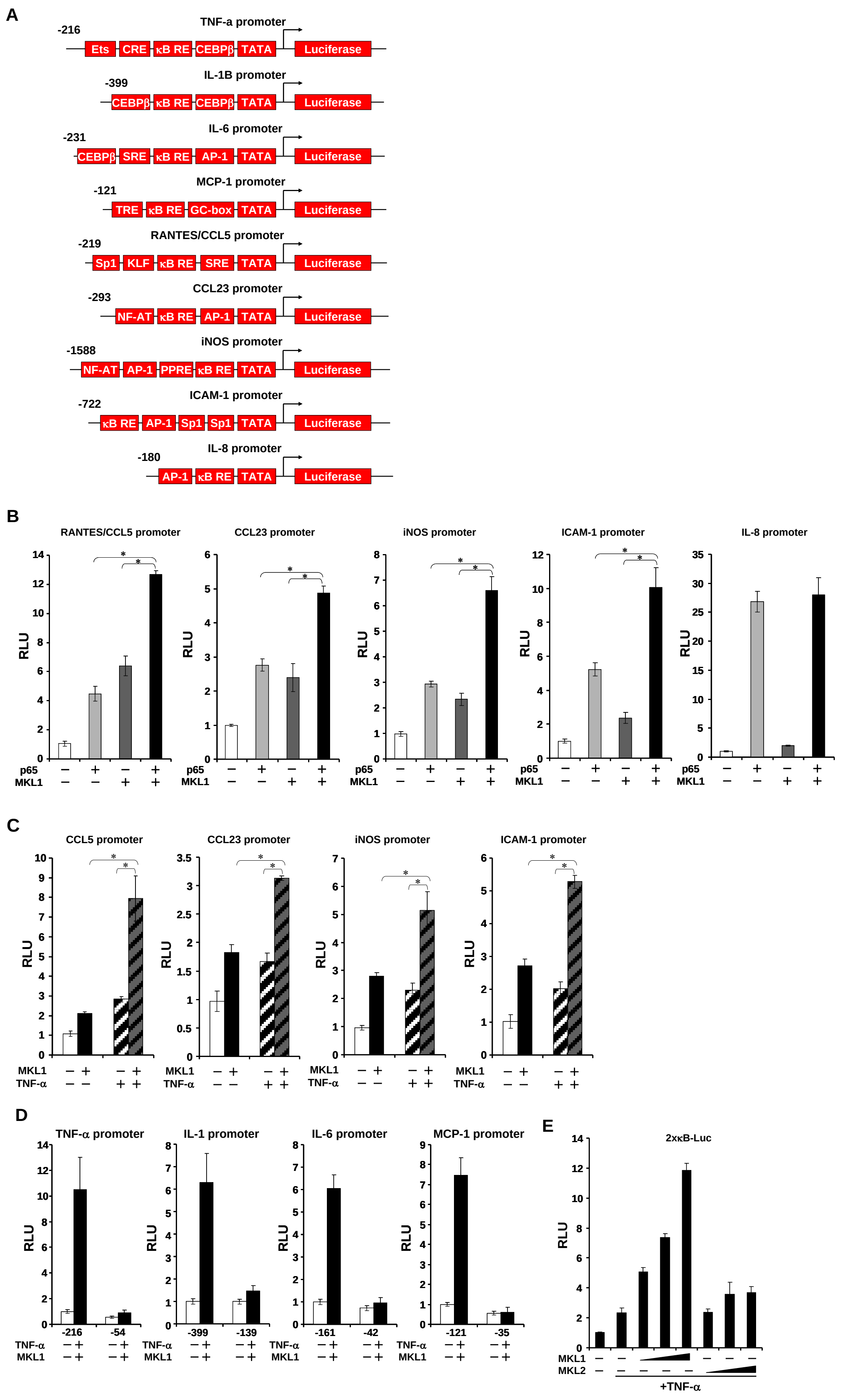
A
TNF-a promoter
-216
Ets CRE κB RE CEBPβ TATA Luciferase
IL-1B promoter
-399
CEBPβ κB RE CEBPβ TATA Luciferase
IL-6 promoter
-231
CEBPβ SRE κB RE AP-1 TATA Luciferase
MCP-1 promoter
-121
TRE κB RE GC-box TATA Luciferase
RANTES/CCL5 promoter
-219
Sp1 KLF κB RE SRE TATA Luciferase
CCL23 promoter
-293
NF-AT κB RE AP-1 TATA Luciferase
iNOS promoter
-1588
NF-AT AP-1 PPRE κB RE TATA Luciferase
ICAM-1 promoter
-722
κB RE AP-1 Sp1 Sp1 TATA Luciferase
IL-8 promoter
-180
AP-1 κB RE TATA Luciferase
B
RANTES/CCL5 promoter
CCL23 promoter
iNOS promoter
ICAM-1 promoter
IL-8 promoter
RLU
p65
MKL1
C
CCL5 promoter
CCL23 promoter
iNOS promoter
ICAM-1 promoter
RLU
MKL1
TNF-α
D
TNF-α promoter
IL-1 promoter
IL-6 promoter
MCP-1 promoter
RLU
-216 -54
-399 -139
-161 -42
-121 -35
TNF-α
MKL1
E
2xκB-Luc
RLU
MKL1
MKL2
+TNF-α

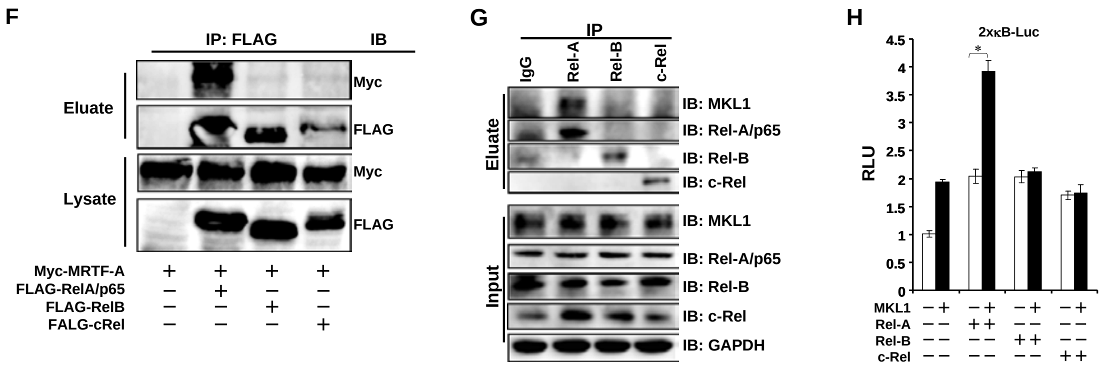

Fig.S1: (**A**) Schematics of the reporter constructs used in this study. CRE, cAMP response element; κBRE, NF-κB response element; SRE, serum response element; TRE, triphorbol acetate response element; PPRE, PPAR response element (**B**) FLAG-MKL1 and V5-p65 were co-transfected into HEK293 cells with indicated promoter constructs. Data are expressed as RLU. (**C**) FLAG-MKL1 was co-transfected into HEK293 cells with indicated promoter constructs followed by treatment with TNF-α (10ng/ml) for 6 hours. Data are expressed as RLU. (**D**) FLAG-MKL1 was co-transfected into HEK293 cells with indicated promoter constructs followed by treatment with TNF-α (10ng/ml) for 6 hours. Data are expressed as RLU. Numbers at the bottom indicate relative position to the transcription start site. (**E**) FLAG-MKL1 or FLAG-MKL2 was co-transfected into HEK293 cells with a generic κB reporter construct followed by treatment with TNF-α (10ng/ml) for 6 hours. Data are expressed as RLU. (**F**) HEK293 cells were transfected with indicated expression constructs. Immunoprecipitation assays were performed with anti-FLAG. (**G**) THP-1 cells were treated with TNF-α for 6 hours. Whole cell lysates were immunoprecipitated with indicated antibodies. (**H**) Indicated expression constructs were co-transfected into HEK293 cells with a generic κB reporter construct. Data are expressed as RLU.

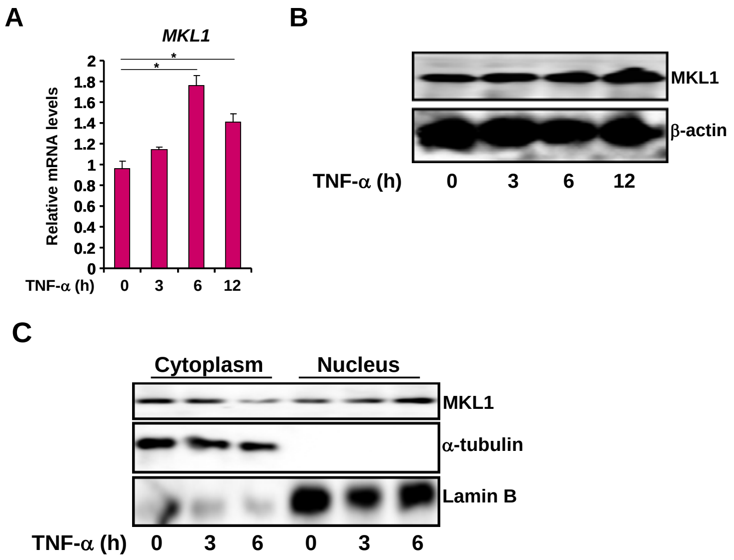

Fig.S2: (**A-C**) THP-1 cells were treated with TNF-α (10ng/ml) and cells were harvested at indicated time points. Expression levels of MKL1 were examined by qPCR (A) and Western (B). MKL1 trans-localization was assessed by cell fractionation followed by Western (C).

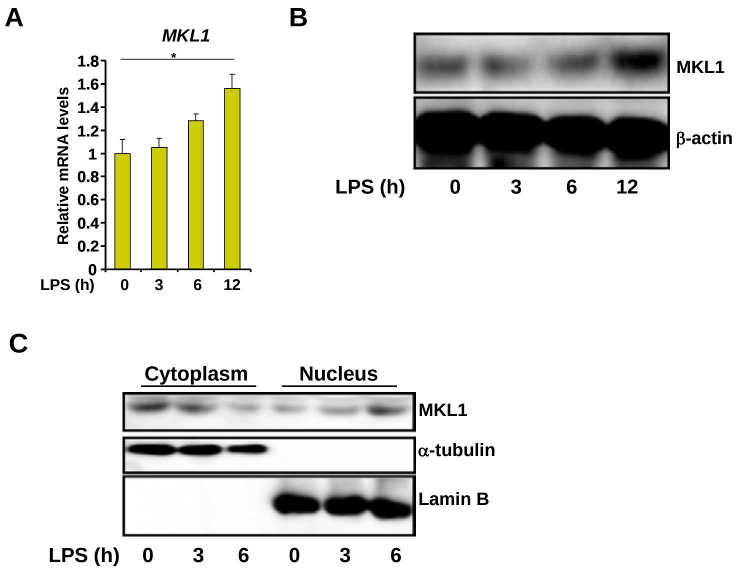

Fig.S3: (**A-C**) THP-1 cells were treated with LPS (100ng/ml) and cells were harvested at indicated time points. Expression levels of MKL1 were examined by qPCR (A) and Western (B). MKL1 trans-localization was assessed by cell fractionation followed by Western (C).

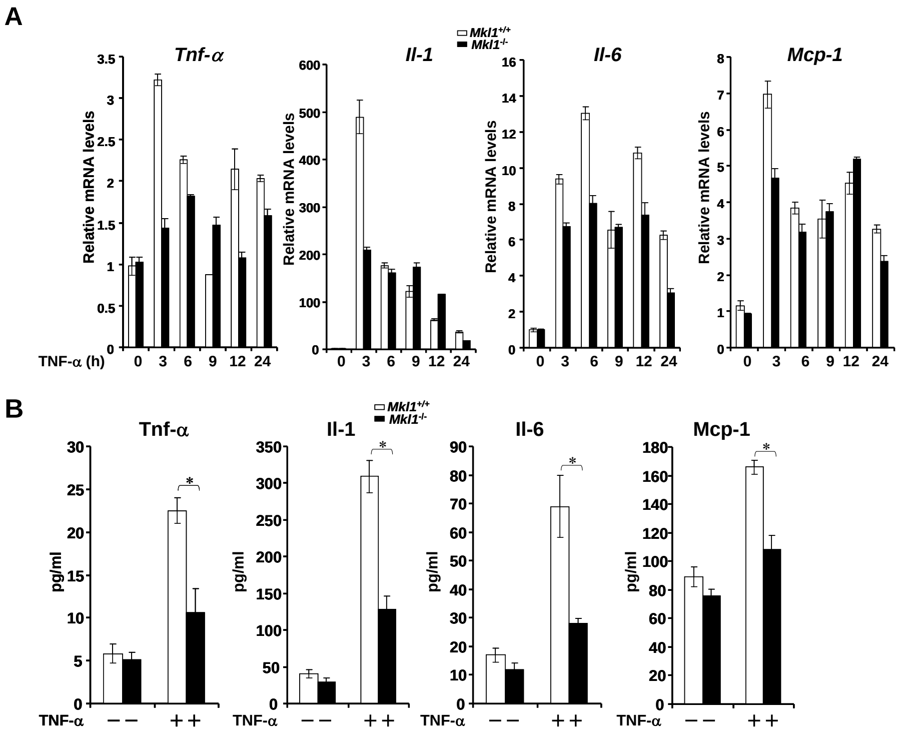

Fig.S4: (**A, B**) Peritoneal macrophages isolated from wild type or MKL1 deficient mice were treated with or without TNF-α (10ng/ml). mRNA (A) and protein (B) levels of pro-inflammatory mediators were measured by qPCR and ELISA.

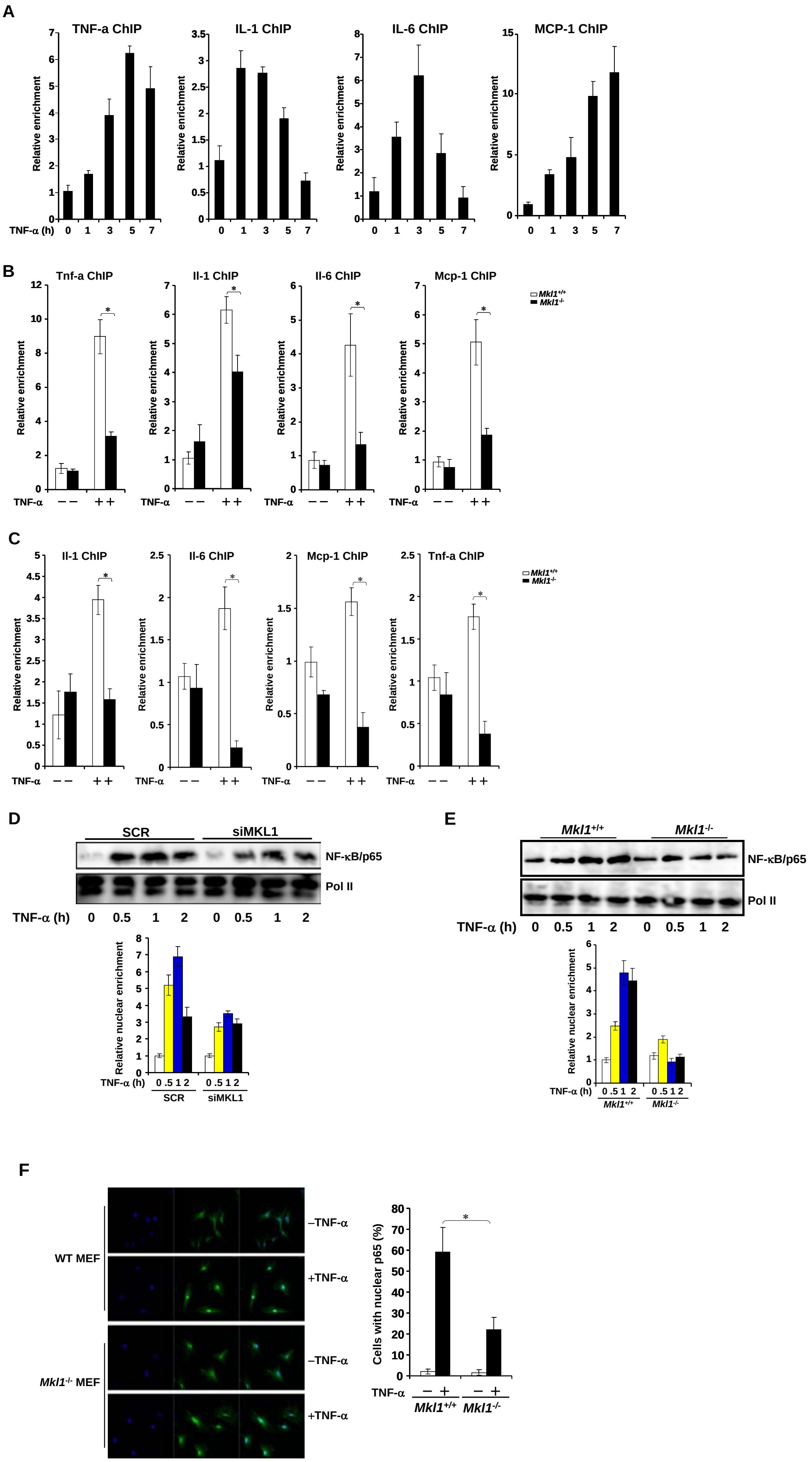
A
TNF-a ChIP
IL-1 ChIP
IL-6 ChIP
MCP-1 ChIP
Relative enrichment
TNF-α (h) 0 1 3 5 7
B
Tnf-a ChIP
Il-1 ChIP
Il-6 ChIP
Mcp-1 ChIP
Mkl1+/+
Mkl1-/-
Relative enrichment
TNF-α − − + +
C
Il-1 ChIP
Il-6 ChIP
Mcp-1 ChIP
Tnf-a ChIP
Mkl1+/+
Mkl1-/-
Relative enrichment
TNF-α − − + +
D
SCR
siMKL1
NF-κB/p65
Pol II
TNF-α (h) 0 0.5 1 2 0 0.5 1 2
Relative nuclear enrichment
TNF-α (h) 0 .5 1 2 0 .5 1 2
SCR
siMKL1
E
Mkl1+/+
Mkl1-/-
NF-κB/p65
Pol II
TNF-α (h) 0 0.5 1 2 0 0.5 1 2
Relative nuclear enrichment
TNF-α (h) 0 .5 1 2 0 .5 1 2
Mkl1+/+
Mkl1-/-
F
WT MEF
Mkl1-/- MEF
−TNF-α
+TNF-α
−TNF-α
+TNF-α
Cells with nuclear p65 (%)
TNF-α − + − +
Mkl1+/+ Mkl1-/-

G

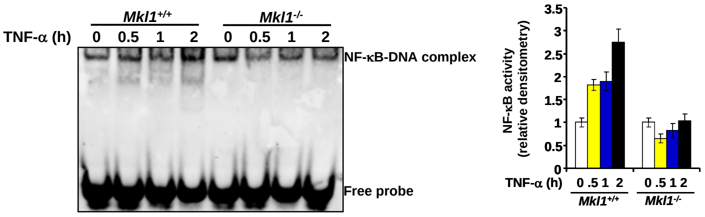

Fig.S5: (**A**) THP-1 cells were treated with TNF-α (10ng/ml). Cells were harvested at indicated time points and ChIP assays were performed with anti-MKL1. (**B**) WT or MKL1 deficient BMDMs were isolated and differentiated as described under *Methods*. Cells were treated with or without TNF-α (10ng/ml) for 6 hours and ChIP assays were performed with anti-p65. (**C**) WT or MKL1 deficient MEF cells were treated with or without TNF-α (10ng/ml) for 6 hours and ChIP assays were performed with anti-p65. (**D**) THP-1 cells were transfected with SCR or siMKL1 followed by treatment with TNF-α (10ng/ml). Cells were fractionated and nuclear p65 was probed by Western. Relative nuclear enrichment of p65 was quantified based on three independent experiments using Image Pro. (**E**) MEFs isolated from wild type or MKL1 deficient mice were treated with or without TNF-α (10ng/ml). Cells were fractionated and nuclear p65 was probed by Western. Relative nuclear enrichment of p65 was quantified based on three independent experiments using Image Pro. (**F**) MEFs isolated from wild type or MKL1 deficient mice were treated with or without TNF-α (10ng/ml) for 1 hour. Intracellular p65 was visualized by immunofluorescence staining. (**G**) MEFs were treated with TNF-α and harvested at indicated time points. DNA binding affinity of p65 was evaluated by gel shift as described under *Methods*. Densitometric analysis of protein-DNA complex formation was performed with Image J based on t three independent experiments.

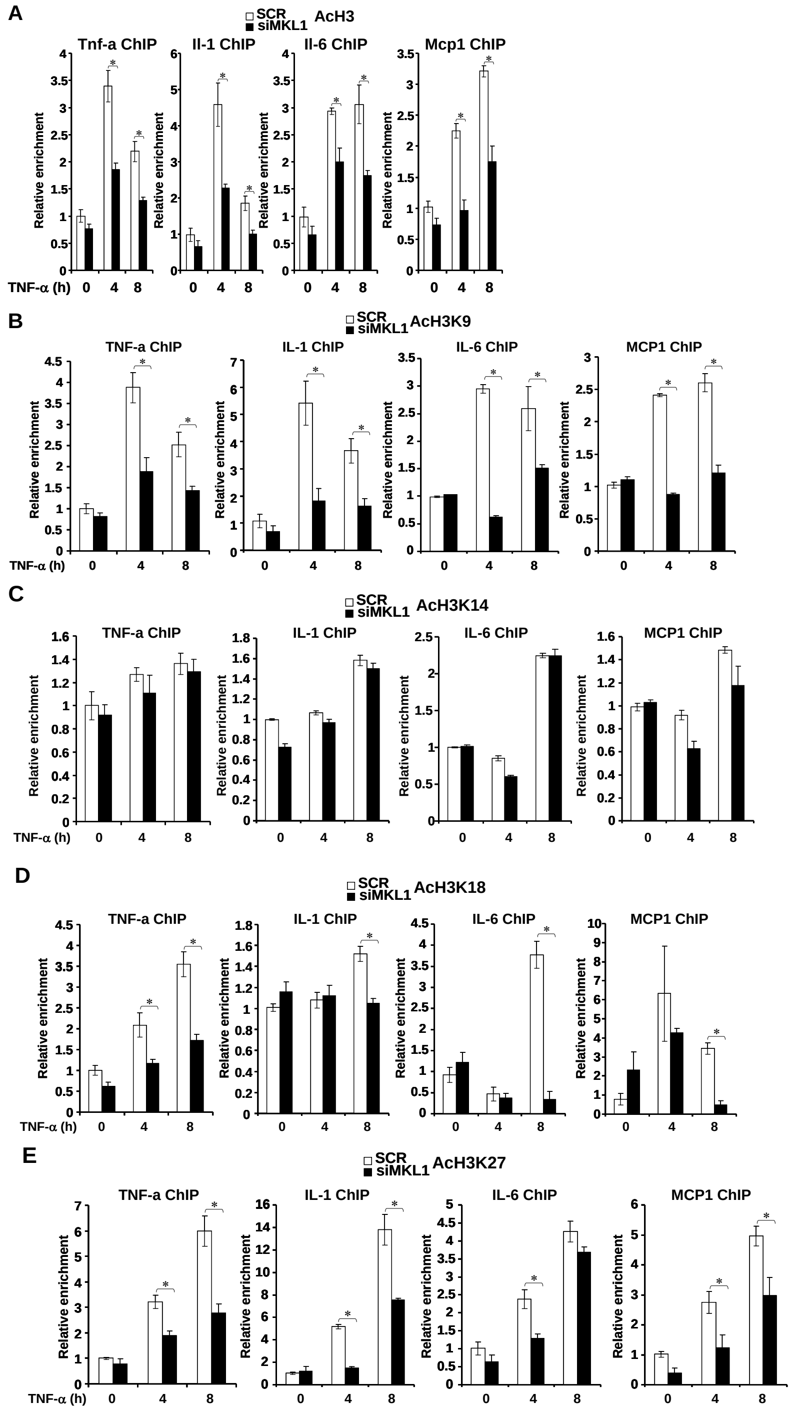

Fig.S6: THP-1 cells were transfected with indicated siRNA followed by treatment with TNF-α□(10ng/ml). ChIP assays were performed with anti-acetyl H3 (A), anti-acteyl H3K9 (B), anti-acetyl H3K14 (C), anti-acetyl H3K18 (D), and anti-acetyl H3K27 (E).

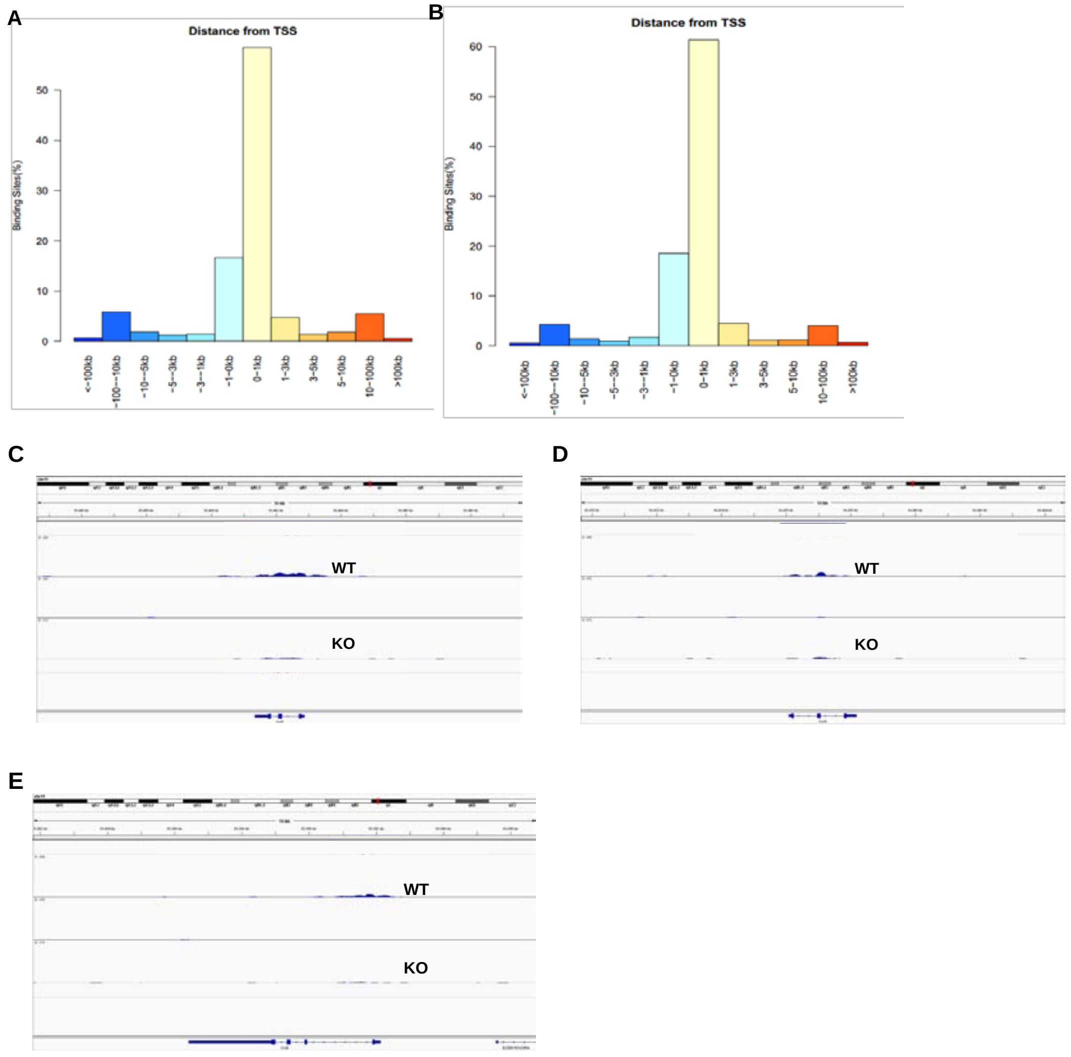

Fig.S7: Wild type (WT) or MKL1 deficient (KO) BMDMs were treated with TNF-α for 3 hours. ChIP was performed with anti-H3K4Me3. (**A, B**) Histogramillustrating the distribution of H3K4Me3 ChIP-seq peaks ± 100kb relative to the TSS of the nearest gene in WT (A) and KO (B) BMDMs. (**C-E**) Graphical view of H3K4Me3 ChIP-seq binding peaks (tag densities) around the TSS of CCL3 (C), CCL4 (D), and CCL9 (E).

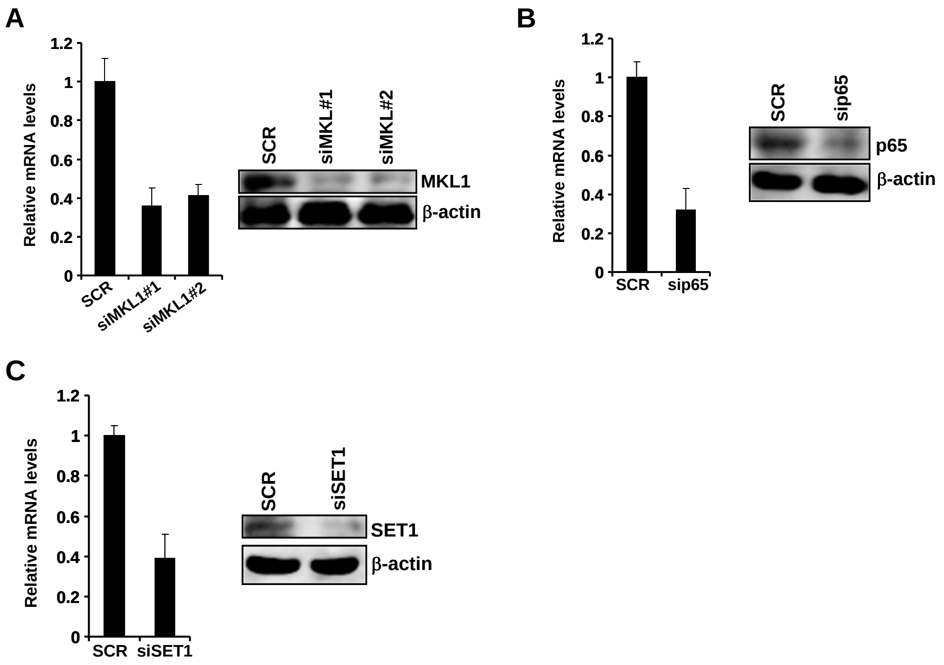

Fig.S8: Validation of small interfering RNAs.

Table I: siRNA sequences

Gene name	siRNA sequences
Human MKL1 #1	GUGUCUUGGUGUAGUGUAA
Human MKL1 #2	CUGCGUGCAUAUCAAGAACAA
Human p65	UGACGUAAAGGGAUAGGGC

Table II: ChIP Real-time qPCR primers

Gene name	Primer sequences
Human TNF-a	Forward: 5’-GCTTCCTCCAGATGAGCTT-3’
	Reverse: 5’-TGCTGTCCTTGCTGAGGGA-3’
Human TNF-a intron	Forward: 5’-AGGATGTGTCTTGGAACTTGGAG-3’
	Reverse: 5’-CTGGCCATGACGTTCTGAGTAT-3’
Human IL-1	Forward: 5’-CTGTGTGTCTTCCACTTTGTCCC-3’
	Reverse: 5’-TGCATTGTTTTCCTGACAATCG-3’
Human IL-1 intron	Forward: 5’-AGTGGAGACGCTGAGACCAGT-3’
	Reverse: 5’-AGTGAGATGCGCAGAACAG-3’
Human IL-6	Forward: 5’-AGAGCTTCTCTTTCGTTCCCGG -3’
	Reverse: 5’-TGTGTCTTGCGATGCTAAAGGACG -3’
Human IL-6 intron	Forward: 5’-ACTAGACTGACTTCTGTAT-3’
	Reverse: 5’- AGAACCAGAATTCGAGT-3’
Human MCP-1	Forward: 5’-CCCATTTGCTCATTTGGTCTCAGC-3’
	Reverse: 5’-GCTGCTGTCTCTGCCTCTTATTGA-3’
Human MCP-1 intron	Forward: 5’-ATCTTCCCTGGTGCTGATCATC-3’
	Reverse: 5’-AATCCCAGTGCTTCTGCCTATAC-3’
